# Comparing serum basal and follicular fluid levels of anti-Müllerian hormone as a predictor of in vitro fertilization outcomes in patients with and without polycystic ovary syndrome

**DOI:** 10.4103/0256-4947.71063

**Published:** 2010

**Authors:** Somayeh Arabzadeh, Ghamartaj Hossein, Batool Hossein Rashidi, Marziyeh Agha Hosseini, Hojjat Zeraati

**Affiliations:** a From the School of Biology, University College of Science, University of Tehran, Tehran, Iran; b From the Imam Khomeini Hospital, Vali-e-Asr Reproductive Health Research Center, School of Medicine, Tehran University of Medical Science, Tehran, Iran; c From the Shariati University Hospital IVF Center, School of Medicine, Tehran University of Medical Science, Tehran, Iran; d From the Department of Epidemiology and Biostatistics, School of Public Health, Tehran University of Medical Sciences, Tehran, Iran

## Abstract

**BACKGROUND AND OBJECTIVES:**

The prediction of in vitro fertilization (IVF) outcomes by anti-Müllerian hormone (AMH) measurement is getting increasing attention from clinicians. This study compares the relationship between serum or intrafollicular AMH levels and IVF outcomes in women with and without polycystic ovary syndrome (PCOS).

**METHODS:**

This prospective study was carried out in two university-based fertility clinics. Serum samples were collected on cycle day 3 and follicular fluid (FF) was collected on the day of oocyte retrieval from 26 women with PCOS and 42 normo-ovulatory controls. AMH levels were measured in the samples using immunoenzymatic assay. The relationship between serum or FF AMH levels and IVF outcomes, including number of oocytes retrieved, oocyte maturation rate, fertilization rate, implantation rate, high quality grade embryo rate, and biochemical and clinical pregnancy rates were further assessed.

**RESULTS:**

Median serum basal AMH and FF AMH levels were significantly higher in the PCOS group as compared to controls, the values being 14.2 ng/mL vs. 3.2 ng/mL (*P*<.001) and 8.2 ng/g protein vs. 4.7 ng/g protein (*P*<.01), respectively. In both groups, serum basal AMH levels showed a positive correlation with number of oocytes retrieved (r=0.323; *P*=.037 in control vs. r=0.529; *P*=.005 in PCOS). In the control group, there was a positive relationship between serum basal AMH levels and percentage of matured oocytes (r = 0.331; *P*=.032) and implantation rate (r=0.305; *P*=.05).

**CONCLUSION:**

Serum basal, and not intrafollicular, AMH levels may be a good predictive factor for quantitative and qualitative IVF outcomes in normo-ovulatory, but not in PCOS patients.

Polycystic ovary syndrome (PCOS) is the most common cause of oligo-anovulation, infertility and hyperandrogenism in women, affecting between 5% and 10% of women of reproductive age worldwide.^1^ PCOS is characterized by an excessive number of growing follicles (2- to 3-fold that seen in normal ovaries) up to the stage of 2 to 5 mm in size (small antral follicles).^1^ In PCOS patients, the selection of one follicle from this increased pool of selectable follicles and its further maturation to a dominant follicle does not occur. The reason for this last phenomenon is unknown, although inhibition of the local effect of follicle-stimulating hormone (FSH) seems pivotal.^2^ In the ovary, anti-Müllerian hormone (AMH) is exclusively produced by the granulosa cells from a wide range of follicles from the primary to the small antral stages of folliculogenesis in the adult rat and human.^3^,^4^ AMH is a glycoprotein of the transforming growth factor-beta (TGF-β) super family, it has become clear that it plays an important role in ovarian function, especially in follicle development and selection.^4^,^5^ AMH seems to inhibit the initiation of human primordial follicle growth^6^ and prevents multiple selection of a dominant follicle.^7^ In addition, it has been shown that AMH is a negative regulator of follicle growth, acting by reducing the sensitivity of follicles to FSH.^8^

One of the main advantages of AMH measurement in IVF treatment instead of the other markers of ovarian reserve may stem from its low inter- and intra-cycle variability. For this reason, AMH could be used as a menstrual cycle-independent marker of ovarian response to controlled ovarian stimulation.^9^ AMH appears to correspond well with antral follicle counts (AFCs) and ovarian response to hyperstimulation in in vitro fertilization (IVF)^10^,^11^ and to be useful for predicting ovarian response in women undergoing IVF treatment.^12^ It has been suggested that serum basal AMH levels represent both the quantity and quality of the ovarian follicle pool during the cycle.^13^–^15^ Lack of success in IVF, indicative of a diminished ovarian reserve, is associated with reduced serum basal AMH concentrations.^16^–^18^ In PCOS, the increase in serum basal AMH level has been shown to be closely related to the degree of menstrual disorder^19^ and with the excess in the 2 to 5 mm follicle number (FN) on ultrasonography (US).^20^,^21^ The role of AMH as a predictive factor of IVF outcomes remains a contentious subject in patients with PCOS. This study sought to determine the relationship between serum basal and intrafollicular AMH levels with the IVF-embryo transfer (ET) outcomes, including number of oocytes retrieved, oocyte maturation rate, fertilization rate, implantation rate, percentage of high quality grade embryo, and biochemical and clinical pregnancy rates in patients with and without PCOS.

## METHODS

This prospective study included 26 infertile women with PCOS, 21 to 37 years of age and with body mass index (BMI) ranging between 17 kg/m2 and 32 kg/m^2^. Infertility due to reduced sperm count or abnormality of sperm was excluded in this group. According to the Rotterdam criteria,^22^ the diagnosis of PCOS was based on the association of at least two of the following three criteria: 1) ovulatory disturbance, mainly oligomenorrhea or amenorrhea; 2) hyperandrogenism, as defined either by hirsutism, seborrhea, and/or testosterone > 0.7 ng/mL and/or androstenedione > 2.2 ng/mL, as measured on day 3 of the cycle; 3) more than 12 follicles in the 2- to 9-mm range in each ovary at US and/or ovarian volume higher than 10 mL. The study was approved by the Institutional Review Board of the Vali-e-Asr Reproductive Health Research Center and Shariati University Hospital of Tehran.

The control group comprised 42 infertile normo-ovulatory women 24 to 42 years of age. All met the following inclusion criteria: 1) both ovaries were present; 2) menstrual cycle length was between 25 and 35 days; 3) there were no current or past diseases affecting the ovaries or gonadotropin or sex steroid secretion; 4) there were no clinical signs of hyperandrogenism; 5) FSH levels were ≤10 mIU/mL on day 3 of the cycle; 6) BMI ranged between 21 kg/m^2^ and 35 kg/m^2^. In all cases, infertility was due to sperm or tubal abnormalities, endometriosis, or was unexplained.

The IVF/ET treatment was initiated with the oral contraceptive pill (OCP) on day 3 or 5 of the cycle and continued with pituitary downregulation by a gonadotropin-releasing hormone (GnRH) agonist consisting of 0.5 mg of buserelin (Suprefact, 1mg/cc, Aventis, Germany) in the mid-luteal phase (day 21) of the cycle, given daily until the day of human chorionic gonadotropin (hCG) administration. On day 3 of the new cycle, ovarian stimulation was started with an injection of recombinant FSH (Gonal-F 75 IU, Serono, Switzerland) or human menopausal gonadotropin (hMG) (Menopur 75 IU FSH: 75 IU LH, Ferring, Germany). The gonadotropin dosage was decided according to the follicular growth, which was monitored by US. The patients were given 10 000 IU of hCG (Pregnyl, Organon, Iran) when at least three follicles became >17 mm. The oocytes were picked 34 h after hCG administration and IVF/ICSI followed by embryo transfer was performed 2 days after oocyte pick-up.

On day 3, the women underwent blood sampling. Sera was separated and frozen in aliquots at –20°C for subsequent analysis. On the day of oocyte pick-up, the FF from more than one follicle (16-20 mm) was gently aspirated and subsequently frozen at –20°C for AMH measurement using an ultrasensitive enzyme-linked immunosorbent assay (Immunotech Coulter, Marseilles, France). The lower detection limit was estimated at 0.1 ng/mL, corresponding to 0.7 pmol/L. The intra- and interassay coefficients of variation were 12.3% and 14.2%, respectively. To avoid possible bias due to FF volume variability, AMH concentrations in the FF were adjusted to its protein content by the Bradford assay. Briefly, a 5× stock of Bradford reagent was prepared as follows: 0.05% (w/v) Bradford reagent + 25% ethanol (v/v) + 42.5% phosphoric acid were mixed together with constant shaking for 2-3 h at room temperature and filtered through Whatman filter paper. All the reagents were provided by Merck and Co. Inc, USA Company. Successive dilutions of BSA (mg/mL) were prepared from a stock solution of BSA 1 mg/mL (Sigma-Aldrich Co, UK) for determination of the standard curve. After 900 µL of 1× Bradford reagent was added to 100 µL diluted FF samples (1/2000), the absorbance of standard and unknown solutions were determined at 595 wavelength using a spectrophotometer (Biorad-Laboratories, Inc. UK). Protein concentration was determined by using linear regression (Microsoft Excel) and the results were expressed as nanogram per gram (ng/g) of protein.

To determine differences between groups, the Mann-Whitney U test was used and correlations were expressed as Spearman correlation coefficients. Multiple logistic regression analysis was used to study independent association among a set of variables. *P*≤.05 was considered to be statistically significant. All statistical procedures were run on SPSS version 13 (SPSS Inc, Chicago, IL).

## RESULTS

In the control group, we found an inverse relationship between serum basal AMH levels and age (r =–0.0323, *P*=.037) and BMI (r =–0.321, *P*=.050), but there was no such a correlation in the PCOS group. Intrafollicular AMH concentration remained unaffected by age and BMI in both groups (data not shown). The main clinical data in the control and PCOS groups are presented in **Table 1**. There were no statistically significant differences between the control and the PCOS group in BMI, number of oocytes retrieved, oocyte maturation rate, percentage of high quality grade embryos, and fertilization or implantation rates. Although, biochemical and clinical pregnancy rates were higher in the PCOS group compared to controls, the difference was not statistically significant.

**Table 1:** Clinical data in control and PCOS group.

	Control (n=42)	PCOS (n=26)	*P*
BMI (kg/m^2^)	27.0 (21-35)	27.0 (17-32)	.370
Oocyte number	9.0 (3-22)	11.0 (2-26)	.066
Oocyte maturation (%)	78.0 (25-100)	71.0 (31-100)	.095
Number of transferred embryos	3.0 (1-5)	3.0 (0-5)	.648
High-quality grade embryo (%)	88.5 (38-100)	82.4 (0-100)	.311
Fertilization rate (%)	62.3 (14-100)	75.5 (50-100)	.128
Implantation rate (%)	5.02	9.19	.265

Values are expressed as median (range) for clinical data. *P*≤.05 was considered significant.

**Figure 1:**
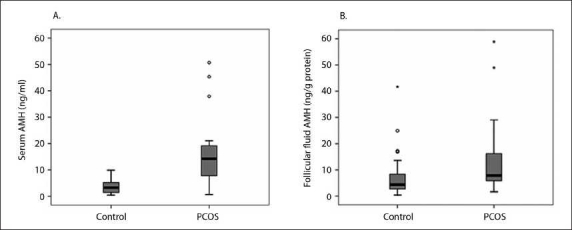
Box plots showing the values of AMH in A) serum (ng/mL) and B) FF (ng/g protein) in control and PCOS groups. Serum and FF AMH levels were significantly higher in the PCOS group than in controls, with *P*<.001 and *P*<.01, respectively.

**Figure 2:**
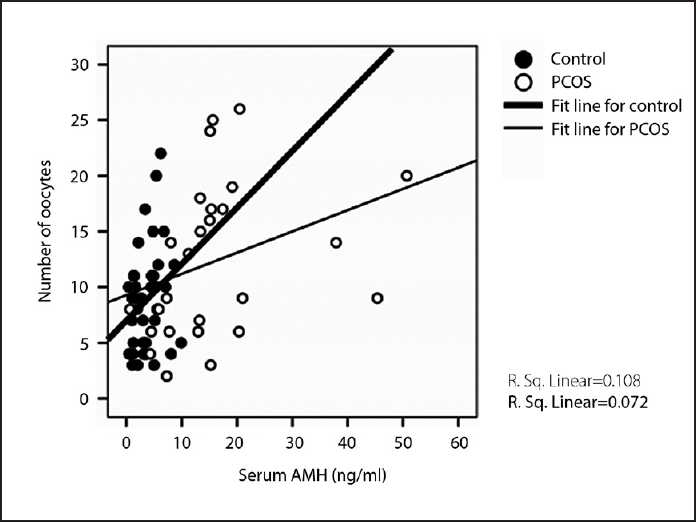
Correlation of serum AMH with oocyte number (r=0.323; *P*<.05 in control vs. r= 0.529; *P*<.01 in PCOS) in control and PCOS groups.

**Figure 3:**
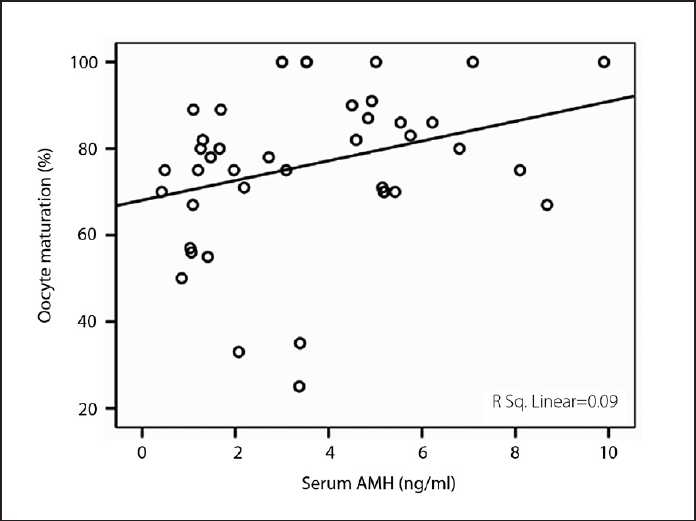
Correlation of serum AMH with oocyte maturation (r=0.331; *P*<.05) in control group.

Median range serum AMH levels were significantly increased in the PCOS group (14.2 ng/mL [0.64-50.7] vs. 3.2 ng/mL [.42-9.90]; *P*<.001) (**Figure 1a**). Similarly, median FF AMH levels were higher in the PCOS group (8.2 ng/g protein (1.6-141.0) vs. 4.7 [0.4-120.0]; *P*=.004) (**Figure 1b**). As shown in **Figure 2**, serum basal AMH levels were positively and significantly correlated with number of oocytes retrieved in both groups (r=0.323; *P*=.037 in control vs. r=0.529; *P*=.005 in PCOS). Similarly, a positive and significant relationship between serum basal AMH levels and oocyte maturation rate (number of matured oocytes×100/number of oocytes retrieved) was observed in control group (r=0.331; *P*<.05), as revealed in **Figure 3**. However, intrafollicular AMH levels remained unaffected by oocyte quantity and quality. The relationship between serum basal and intrafollicular AMH levels with IVF/ET outcomes for both groups is shown in **Table 2**. Fertilization rate (number of embryos forming×100/injected oocyte number) was unrelated to serum basal and intrafollicular AMH levels in both groups. In the control group, the implantation rate (total number of gestation sacs×100/total number of embryo transferred) showed a positive and significant relationship with serum basal AMH level. Day 2 cleavage-stage embryos were graded 1-4 by the embryologist according to established morphological criteria, with good quality embryos (grades 1-2) being preferentially transferred rather than those of poorer quality (grades 3-4). High-quality grade embryo (number of the embryos forming (grade 1 + grade 2) ×100/total number of embryo)remained unaffected by both serum basal and intrafollicular AMH levels in both groups.

**Table 2:** Relationship between AMH levels in serum (ng/mL) and FF (ng/g protein) with IVF/ET outcomes.

	Control (n=42)		PCOS (n=26)	
	r	*P*	r	*P*
Serum AMH, fertilization rate	−0.247	0.115	0.053	.796
Serum AMH, implantation rate	0.305	0.050^a^	−0.299	.138
Serum AMH, high quality grade embryo	−0.108	0.494	0.150	.474
FF AMH, fertilization rate	−0.035	0.827	−0.111	.589
FF AMH, implantation rate	0.246	0.117	0.134	.513
FF AMH, high quality grade embryo	0.091	0.568	−0.309	.133
FF AMH, oocyte number	−0.118	0.456	−0.162	.429
FF AMH, oocyte maturation	0.275	0.078	0.186	.363

*P*≤.05 was considered significant.

a *P*≤.05

The serum basal AMH level, but not intrafollicular AMH level-showed a positive correlation (*P*=.054) with biochemical pregnancy rate in the control but not in the PCOS group. No significant correlation was observed between serum or intrafollicular AMH levels and clinical pregnancy rate (gestational sac as observed at US after 7 weeks of amenorrhea) in both groups. Moreover, when assessed by multiple logistic regression analysis, none of the assessed outcomes had a significant effect on biochemical/clinical pregnancy rates.

## DISCUSSION

The aim of this study was to compare serum basal to intrafollicular AMH to assess their usefulness for predicting quantitative and qualitative IVF outcomes in patients with and without PCOS. In summary, the results of this study clearly indicate that serum basal and intrafollicular AMH levels are significantly higher in women with PCOS than in controls. Furthermore, the results suggest that serum basal AMH level is predictive of oocyte quantity and quality, as is evident by the higher oocyte maturation rate in control than in PCOS patients. On the other hand, intrafollicular AMH level could be a good marker for oocyte quantity but not their quality. So far as is known, this study is the first to describe a significant link between serum basal AMH levels and implantation rate in normo-ovulatory women. These results strengthen the body of evidence supporting the use of day 3 serum AMH measurements for the prediction of pregnancy outcomes during IVF treatment in normal control but not in PCOS patients.

AMH appears to correspond well with antral follicle counts (AFCs) and ovarian response to hyperstimulation in IVF.^10^,^11^,^23^ The increased serum basal AMH concentrations in PCOS have been explained by the increased number of small ovarian follicles responsible for AMH secretion.^20^ We also demonstrated that FF AMH levels are greater in women with PCOS compared to normo-ovulatory women, which may suggest increased per follicle AMH secretion in PCOS.^24^

All the studies (including this report) have confirmed the utility of early follicular phase serum AMH concentration as a predictor of the number of retrieved oocytes, which could support the concept of a direct relation between serum AMH levels and AFCs in an IVF cycle.^9^,^10^,^15^,^18^,^19^,^24^–^26^ However, data on serum or FF AMH levels in relation to oocyte quality are still contradictory. Like our report, one study demonstrated a positive and significant correlation between serum basal AMH levels and proportion of oocyte in metaphase II.^27^ On the other hand, a recent study showed no correlation between serum day 3 AMH levels and oocyte maturation.^28^ Ebner showed that serum basal AMH values between 1.66 and 4.52 ng/mL were associated with high-quality oocytes, but higher serum AMH levels could be harmful for oocyte maturation.^16^ Moreover, serum AMH levels at ovulation triggering day were more closely related with immature rather than mature oocyte proportion.^29^

We have shown here a nonsignificant inverse relationship between intrafollicular AMH levels and oocyte quantity in both PCOS and control groups. It has been postulated that AMH decreases the sensitivity of growing follicles to FSH and final selection of dominant follicles.^8^,^30^ Thus, it is tempting to speculate that high intrafollicular AMH levels may have a negative effect on oocyte quantity. Until now, the relationship of FF AMH levels with oocyte quality was uncertain. Cupisti et al found that AMH levels in individual follicles were inversely correlated with the maturation and developmental potential of oocytes.^31^ On the contrary, Takahashi et al observed that oocytes were more likely to be fertilized when their follicle was able to produce high levels of AMH, as FF AMH levels from follicles with fertilized oocytes were more than three times higher than from follicles with non-fertilized eggs in normo-ovulatory women.^32^ However, Mashiach et al demonstrated that in PCOS patients, the proportion of matured oocytes, as well as the success of fertilization, did not correlate with FF AMH.^33^ Indeed, excess in FF AMH was shown to have harmful consequences on oocyte quality and final maturation.^34^ On the whole, it seems that determination of serum or intrafollicular AMH cutoff values could be of great importance for understanding the relationship with oocyte quality.

Most studies (including this report) have shown that fertilization rate, high quality grade embryo, and clinical pregnancy rates are unaffected by AMH levels.^32^,^35^–^38^,^21^,^25^ In addition, trying to identify cutoff values for serum basal and FF AMH levels, Fanchin et al showed that intrafollicular AMH level is a better predictor of implantation and clinical pregnancy rate than serum basal AMH levels in normo-ovulatory patients; however, this was not confirmed by other studies.^35^ We have shown a correlation between serum basal AMH levels and implantation rate in normo-ovulatory patients, which needs to be further examined in a larger study.

In conclusion, serum basal AMH levels could be a good marker for oocyte number in both PCOS and infertile normo-ovulatory women. Further, we suggest that serum AMH level may be a predictive marker of oocyte maturation and implantation rate in normal women. The lack of consistency between AMH levels and qualitative IVF outcomes in PCOS patients may be influenced by the presence of differing PCOS phenotypes, with either primary ovarian dysfunction or insulin and obesity being the major contributor to reproductive dysfunction.
